# Oral Health Policy and Improvement Strategies in Oklahoma

**DOI:** 10.3389/froh.2022.850602

**Published:** 2022-03-24

**Authors:** Michelle Dennison, Julie Seward

**Affiliations:** ^1^Oklahoma City Indian Clinic, Oklahoma City, OK, United States; ^2^Southern Plains Tribal Health Board, Oklahoma City, OK, United States

**Keywords:** oral health, dental health aide therapist, health policy, sovereign nations, American Indian or Alaska Native, dental therapy

## Abstract

Oklahoma an ethnically, financially and geographically diverse population has unique oral health care challenges. These challenges include poor overall oral health, inadequate oral health coverage, significant physical access to care barriers and a shortage of oral health care workers. Just as the oral health care barriers are diverse, so are the potential solutions. Potential solutions include efforts at all levels of government, innovations of health care delivery and recognition of the unique needs of Oklahoma American Indian population. Potential strategies address each of these opportunities and recognize both the short and long term needs of Oklahoma oral health.

## Introduction

Despite being a large contributor to overall health and wellness with linkages to chronic disease such as diabetes, metabolic syndrome and cardiovascular disease [1], oral health care is an often overlooked and underprovided service in Oklahoma. Oklahoma's oral health statistics reflect a population with high rates of childhood tooth decay, adult tooth loss and absence of regular oral health appointments primarily due to cost. Root causes include financial and physical barriers and widespread oral health care professional shortages. Only 72% of Oklahoman children have received one or more dental visits compared to 80% nationally, and 66% of third graders have experienced tooth decay compared to only 52% of the national population [2]. The statistics are no better for adults: 60% of Oklahoman adults visited the dentist in 2019 compared to 68% nationally, and only 35% of pregnant women had their teeth cleaned during pregnancy compared to 46% nationally [2]. Additionally, 42% of Oklahomans aged 65 or older have lost at least six teeth to decay or gum disease [3]. Inequities in oral health access and outcomes for Oklahomans show some populations experience greater barriers than others. Cost is cited as the main reason for avoiding dental care [4, 5] with low income adults and children in Oklahoma being less likely to see a dentist and low-income older adults are also more likely to experience tooth loss (Table 1) [6, 7]. Much of the oral disease Oklahomans experience is preventable [8]; however, accessing the necessary oral health cares can be expensive and difficult.

**Table 1:** Oklaoma oral health statistics [2–7].

**Children: received one or more dental visits**	
Oklahoma	72%
Nationwide	80%
**Children: tooth decay experience**	
Oklahoma	66%
Nationwide	52%
**Adults: visited a dentist in the past 12 months**	
Oklahoma	60%
Nationwide	68%
**Pregnant women: received a dental cleaning**	
Oklahoma	35%
Nationwide	46%
**Adults 65 or older: lost at least six teeth due to tooth decay or gum disease**	
Oklahoma	42%

## Policy Options And Implications

### Oral Health Are Access Barriers: Financial

Approximately 23% of American Indian or Alaska Native (AI/AN) people live in poverty compared to 10.5% for the US general population, and in Oklahoma, poverty affects 19.3% of AI/AN peoples [9]. Currently, 29% of Oklahoma's population receive Medicaid benefits. AI/AN people living in Oklahoma make up 11% of the total Medicaid enrollment for the state [10]. In 2020, prior to the recent Medicaid expansion, 533,000 Oklahomans were uninsured, the second highest rate in the nation, leaving many Oklahomans without realistic options for dental care [11]. Of insured patients, 14.6% of adults avoid oral health care due to non-covered costs [12]. For the uninsured, 30% of adults do not receive the dental care they need due to cost [4]. A recent report outlining the challenges and potential solutions for oral health, show 68% of respondents stated cost as the biggest barrier for improved oral health in Oklahoma communities [13].

### Oral Health Care Access Barriers: Physical

Physical access to oral health care is also a burden as Oklahoma, a largely rural state, encompasses 70,000 square miles and 4 million residents, 45% of whom live in rural areas. According to data published in 2017, 66% of the AI/AN population live in rural Oklahoma [14]. Further, many rural areas in Oklahoma do not have adequate oral health providers. According to the Health Resources and Serviced Administration (HRSA), 1.3 million Oklahomans live in counties considered dental health professional shortage areas, further hindering access to care [15]. The lack of oral health providers in these areas leave residents with limited options other than to travel long distances for care [16]. Those with unreliable transportation are more likely to receive inadequate or no care.

Oklahoma does not have the number of oral health providers needed to support the population, with 55.3 dental care providers per 100,000 people compared to 61.2 nationally [12]. Additionally, fewer than half of dentists in Oklahoma accept Medicaid [17]. Those who face health care expense obstacles are even more challenged when that provider is physically distant or inaccessible.

### Oral Health Care Access Barriers: Workforce

In both private and Tribal health systems employers are challenged to fill dental provider positions. The national workforce is estimated to need over 10,000 additional dental health professionals to meet the current population's needs [18]. As a result, over 80% of dental practices report recruitment challenges [19]. A recent search of the Indian Health Service (IHS) open positions database shows an ongoing trend of dentist, hygienist and assistant vacancies, with multiple open positions at some practices. This indicates a true and unacceptable deficiency in the ability to provide adequate care to the American Indian population [20]. Oklahoma's Indian health care delivery systems include federally managed clinics, urban Indian organizations and Tribally operated health care systems, referred to as I/T/Us. Rural Oklahoma has 45 I/T/U systems, which serve over 380,000 patients statewide [21]. As mentioned, staffing shortages within the I/T/Us contribute to delayed care and inadequate funding often limit services resulting in high rates of underfunded referrals to outside providers [22]. As mentioned the lack of health care providers is not unique to the I/T/U system and is felt across service delivery models. Specific root causes include lack of sustainable funding to recruit and retain providers and the high administrative burden now placed on health care providers, which ultimately reduces patient care productivity.

### Oral Health Care Access Solutions: Financial

After the Medicaid expansion on July 1, 2021, an additional 250,000 Oklahomans, most of which were adults, enrolled in Medicaid [10]. As mentioned, ~11% of the Oklahoma Medicaid population is American Indian [10]. Also in 2021 the Medicaid oral health coverage benefit which was previously limited to children, was expanded to adults [23, 24]. The cumulative effect of Medicaid expansion and addition of adult oral health should provide new and robust services to the adult population, which may partially solve the previous oral health care coverage issues. However, increased utilization and demand for preventative services are expected and may put further strain on the system.

Fortunately, with the latest Medicaid budget changes, the I/T/U systems can expect a significant increase in revenue for adult services already provided. This change will directly improve the services available for Medicaid recipients. It may indirectly improve oral health services by increasing clinical capacity through increased funding to I/T/Us as well. Oral health insurance coverage options and equitable policies should continue to be explored, as I/T/U systems may still find it difficult to provide the services needed and meet optimum staffing levels.

### Oral Health Care Access Solutions: Physical

In the absence of an increase in oral health care providers in Oklahoma, innovative care delivery options should be explored. The COVID-19 pandemic forced health care providers to transition some services to telehealth. This trend resulted in significant improvements in access to health care, including those with transportation issues and physical limitations. To support the benefits of telehealth, the Oklahoma State Legislature has defined telehealth modalities and coverage mechanisms [25], as well as basic teledentistry [26]; and the American Dental Association has issued a position statement supporting teledentistry to provide patient care and education [27]. To further maximize teledentistry in Oklahoma, legislation and regulation to solidify insurance coverage and reimbursement requirements are necessary.

### Oral Health Care Access Solutions: Workforce

Addressing deficiencies in access to care and provider availability, especially in the overburdened I/T/U system, requires examination of the workforce and care delivery. Expanding the services that dental assistants and hygienists can perform and authorizing a new provider type, the dental therapist (DHAT), might provide necessary care to more rural and Tribal populations. DHATs are licensed mid-levels that provide some of the most common dental procedures, such as exams and fillings. DHATs work under the supervision of a dentist [28], and are able to bring care directly to people including in schools and nursing homes, Tribal communities and rural areas [28]. DHATs have been utilized since 2005 and are currently authorized to work, in varying capacity, in 12 states [28]. While DHATs have been working globally for a century, the dental therapy profession was brought to the US by Alaskan Tribal leaders seeking to improve oral health in their communities. Under federal authority, these leaders created a program that focused on educating Alaska Natives to deliver the care their communities needed most. By focusing the scope of DHATs on a small set of commonly needed procedures, they created an education program that was accessible and affordable. The result has been a sustainable, culturally appropriate dental therapy program that creates well-paying jobs in underserved communities while increasing access to dental care and improving oral health outcomes [29]. In 2015, the US Commission on Dental Accreditation (CODA), the organization responsible for accrediting education programs for dentists and dental hygienists, issued dental therapy standards [30]. Having CODA accredit dental therapy education programs ensures the providers are educated to the same high standards as other dental providers and takes the burden off individual states to set the education requirements for dental therapists [30]. Dental therapists, supervised directly by a licensed dentist, have been shown to improve access to care for underserved communities and overall oral health [29, 31]. Transferring lower-level dental procedures to DHATs would allow dentists to focus on more complex care, making the dental team more efficient and accessible [32]. Employing dental therapists means lower employment cost and allows community health centers and I/T/U clinics to stretch limited budgets and makes private practice dentists more profitable [33]. Because dental therapy education is shorter and less expensive and can be offered at Tribal or community colleges, dental therapy has created accessible workforce pipelines from and to underserved communities.

### Access to Care Solutions: A Community Perspective

**Figure 1:**
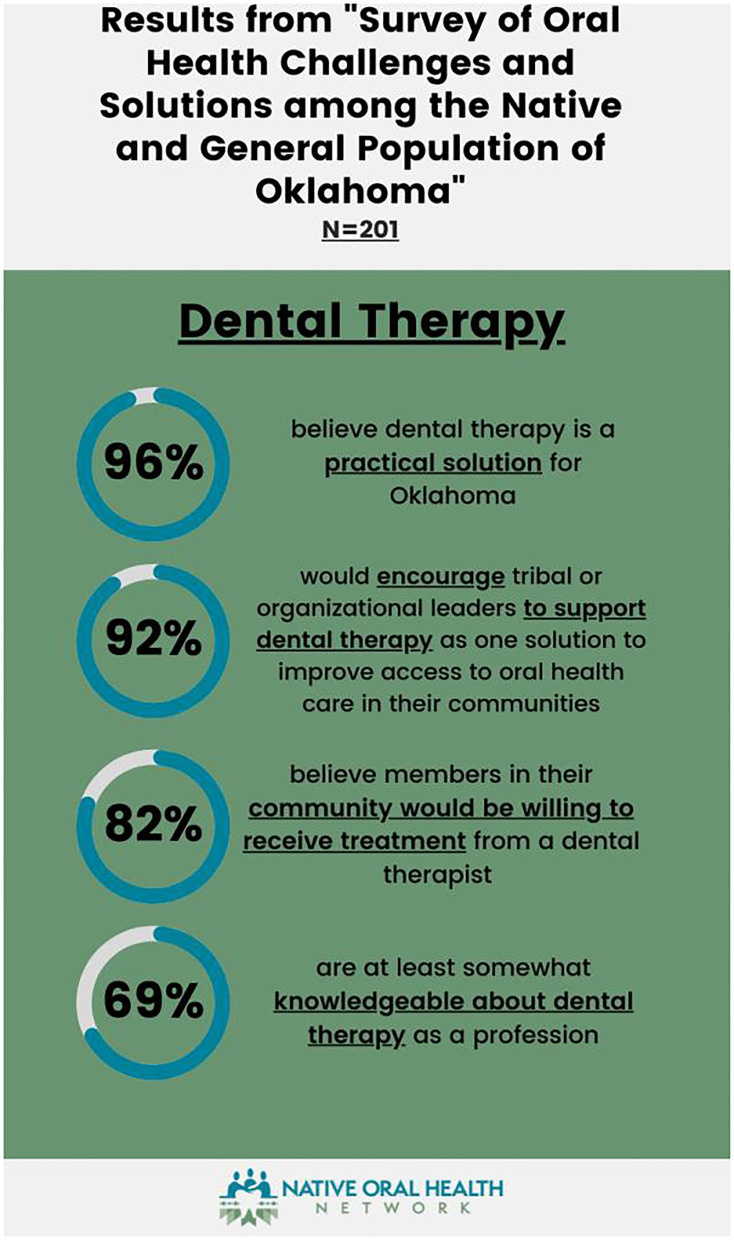
Oral health challenges survey results. Image courtesy of the Native Oral Health Network.

Oral health care deficiencies are felt beyond the oral health care professions. A survey of AI/AN and the general population found that a lack of rural oral health care services and covered benefits were of highest concern. Approximately 61% of respondents felt expanding the scope of practice for Oklahoma dental hygienists would help address the provider shortage, and 58% want to expand the oral health care profession types in Oklahoma. The addition of a DHAT was supported by 96% of respondents; however, 31% of the respondents voiced a need for advocacy to improve understanding of the profession (Figure 1). These outcomes show a general awareness of the Oklahoma oral health care status and need for advocacy for oral health care funding, expanded oral health care professions and scopes of work [13]. Partnerships with Oklahoma tribes are essential to identify community-based data collection methods and community-informed approaches that encompass the breadth of the disparities.

### Federal Level Solutions

To solve oral health care professional shortages, discussions around expanding the scope of work for dental hygienists and/or adding new levels of oral health care professionals are necessary. The Indian Health Care Improvement Act (IHCIA) [34], the authority for the health care provision to AI/AN people, provides guidance for increasing the number of oral health care professionals. The IHCIA specifically identifies DHATs as a profession that Tribes can utilize to expand access to care. However, the IHCIA requires that Tribes outside Alaska not employ DHATs under the Community Health Aide Program unless the Tribe is in a state with a dental therapy licensing law. Tribes and national organizations such as the National Indian Health Board (NIHB) are advocating for Congress to remove this restriction, as the requirement infringes Tribal sovereignty and because DHATs practicing under the Community Health Aide Program receive federal certification, not necessarily state licensure [34]. Urban Indian clinics are also specifically excluded from accessing this benefit within the IHCIA [34], thus limiting the benefit to the AI/AN patients accessing Tribal health services. To overcome this regulatory barrier state legislation is required.

States have the flexibility to determine what dental benefits are provided to adult Medicaid enrollees. There are no minimum requirements for adult dental coverage. Making adult dental services mandatory in Medicaid would expand access to dental care for millions of low-income AI/AN peoples outside the I/T/U system while improving the ability of I/T/U systems to meet their patients' oral health needs. The optional status of Medicaid adult dental coverage means that states can take away these benefits at any time. Medicaid adult dental benefits are often subject to state budget cuts during economic downturns, especially in states with more comprehensive coverage. This means that states may offer different oral health coverage to people in different eligibility categories, such as during pregnancy or people with disabilities. This narrow definition of benefits can be confusing for enrollees and oral health providers, especially when covered services change with state budget fluctuations [4]. Because AI/AN people covered by Medicaid are not required to pay premiums or enrollment fees, standardizing Medicaid adult dental benefits could significantly increase access to affordable dental care for the hundreds of thousands of AI/AN adults already covered by Medicaid.

### Tribal Level Solutions

Tribal health systems, by virtue of sovereignty, have the option to expand and self-regulate health care professionals within their employment. Tribes, in conjunction with HRSA and following CODA standards [28], may exercise Tribal sovereignty and train, license and employ dental therapists. Precedent for this process has been set by the Swinomish Indian Tribal Community of Washington state [35]. However, this solution does not extend to non-Tribal Federally Qualified Health Centers, urban clinics, or private practices; therefore, it does not comprehensively address the oral health care needs in Oklahoma or the AI/AN population. Excluding Tribal sovereignty, the best route for expanding the types of oral health care professionals in Oklahoma is a change in the statute to authorize DHATs to practice statewide.

### State Level Solutions

Within the state of Oklahoma, the oral health policy landscape is largely directed by the Oklahoma Dental Act [36], which is enforced by the Oklahoma Dental Board. Currently, dentists and dental hygienists are recognized as oral health care providers in Oklahoma. Changes to this statute, such as the addition of a mid-level oral health care provider or changes in scopes of practice, must be approved by the Oklahoma State Legislature and the Oklahoma Dental Board [36]. The Oklahoma Dental Board currently consists of nine dentists, one dental hygienist and two at-large members [37]. Sixty-four percent of the current Oklahoma Dental Board represents urban communities, and none represent Tribal or Urban Indian clinics. Due to this lack of diverse representation, the current board makeup presents challenges for AI/AN health care advocacy. Oral health care in Oklahoma can be improved through the unified efforts of Tribes, urban Indian organizations, dental associations and others and state-level legislation that targets access to care barriers and benefits all oral health providers.

## Actionable Recommendations: Strategies For Improving Ai/An Oral Health Care In Oklahoma

To improve Oklahoma AI/AN oral health, solutions are required that will benefit the needs of all citizens regardless of where they live or seek care. As with any complex health issue, these solutions include creative and all-inclusive thinking, broad education, and strong advocacy. Strategies designed to address each of the deficiencies discussed above are outlined below and were prioritized for feasibility, benefit applicability and potential for uptake. To fully overcome state oral health barriers, effective and all-inclusive state legislation is required.

### Financial Access to Care

1) Advocate for standardized, mandatory Medicaid adult dental benefits.
2) Advocate for defined coverage and reimbursement parity for teledentistry services for all payers.

a. Leverage recent Medicaid oral health inclusion language to support the need for expanded benefits.

3) Advocate for the broad uptake and utilization of available oral health Current Procedural Terminology (CPT) codes within all I/T/U health systems.

### Physical Access to Care

1) Advocate for technical support for the uptake and utilization of teledentistry services.
2) Advocate for broad coverage and reimbursement for transportation options to oral health care providers.
3) Advocate for the continued and expanded recruitment and retention of oral health care professionals within I/T/U health systems.

### Workforce Deficiencies

1) Advocate for addition/expansion of scopes of practice for oral health care providers.
2) Provide education about cost savings, revenue streams and services of mid-level oral health care providers to community/professional partners, medical and dental associations, and policy authorities.

a. Provide education about the rigor and requirements of CODA educational standards for dental therapy programs.
b. Provide education and support for state-led efforts for addition/expansion of scopes of practice for oral health care providers.

3) Support I/T/U representation on the Oklahoma Dental Board.
4) Advocate for Tribal-level dental therapy programs, encouraging the use of Tribal sovereignty to implement training programs, licensing and regulation.
5) Advocate for the continued and expanded recruitment and retention of oral health care professionals within I/T/U health systems.

## Conclusion

Oral health is critical for overall health and wellness. Improving access to dental care by adopting policies that increase access can help decrease medical costs associated with chronic conditions that continue to put Oklahoma in the top rankings for various leading causes of death [38]. Timely advances in Oklahoma's oral health policies, including the adult Medicaid oral health benefit, increases patient-driven demands for oral health care services. For facilities that receive the Office of Management and Budget (OMB) rate, significant increases in revenue are expected. If reinvested back into oral health care services, this additional revenue will increase the depth and breadth of services provided resulting in reduction to some of the access-to-care barriers discussed. However, improved financial access to care may further strain an already understaffed workforce. Further discussion of solutions to workforce shortages is necessary now, as they are predicted to become more significant in the near future. Solutions for this problem require a unified voice and open-minded discussions between Tribal and non-Tribal entities, dental associations and legislators to support Oklahoma's unique needs.

## Author Contributions

All authors listed have made a substantial, direct, and intellectual contribution to the work and approved it for publication.

## Funding

Oklahoma Shared Clinical and Translational Resources Center (grant subaward number: RS20180476-47A1). National Indian Health Board 2021–2022 Tribal Oral Health Initiative Dental Health Aide Therapy Education and Outreach Grant.

## Conflict of Interest

The authors declare that the research was conducted in the absence of any commercial or financial relationships that could be construed as a potential conflict of interest.

## Publisher's Note

All claims expressed in this article are solely those of the authors and do not necessarily represent those of their affiliated organizations, or those of the publisher, the editors and the reviewers. Any product that may be evaluated in this article, or claim that may be made by its manufacturer, is not guaranteed or endorsed by the publisher.
